# Post-Mortem Caloricity of Yellow Fever

**Published:** 1845-10

**Authors:** Bennett Dowler

**Affiliations:** New Orleans


### RECORD OF MEDICAL SCIENCE.

### *Post-Mortem Caloricity of Yellow Fever.*

By BENNETT DOWLER, M.D. of New Orleans.

CASES.—*The morbid caloricity, very great and uniform, from one to two hours after death, dissipates itself equally and simultaneously in the epigastrium and thigh.* October 2d, noon; room 84°. I. S., a German butcher, aged 29; last from Havre; resident eleven days; sick nine days; dead one hour; neck quite rigid, arms and legs moderately so; corneæ brilliant and natural, &c.; [at the commencement of the experiments, both pupils were dilated—one eye was closed—its pupil remaining stationary; the other pupil, after exposure to light contracted in an hour; all the while the covered corneæ was natural, while the exposed one, from desiccation, became dim or glassy;] axilla in 20 minutes, 106°; the thigh in 10 m. 106°; the left chest in 15 m. 106°; the thigh in 5 m. 106°; the epigast. in 10 m. 106°—5 m. 105°; thigh in 5 m. 105°—10 m. 104°—three hours dead.

*The thigh gives the maximum—the whole mass at about* 106°, *as long as observed.* N. E., born in Italy, aged 25; last from Marseilles; resident seventeen days; sick six days; dead twenty minutes; [abdomen concave, recti muscles contracted into hard knotty ridges; neck rigid; limbs flexible; the arm was extended—an axe or large hatchet being tied in the palm, the flexors of the fore-arm were struck with the inferior edge of my extended hand; the subject raised the fore-arm, carrying the weight, about three lbs., several times, striking the same near the centre of the trunk, at several places from the umbilicus to the upper end of the sternum, &c.—See Note.]—axilla 8 minutes, 106°; perineum (closing the limbs) 5 m. 104°; axilla 5 m. 106°—6 m. over 106—3 m. same; thigh 4 m. 105°—5m. 106½°; epigast. 4 m. 106°—2 m. 106° and over; left chest 6 m. 105°; thigh 9 m. 106½°; left chest 5 m. 104°; epigast. 5 m. 106°; thigh over 106°; at about two hours after death, when the experiments were abandoned.

*Two hours after death the heat continues to augment—soon reaches its maximum, continuing stationary, except a slight oscillation, for two hours—at the sixth hour, the law of atmospheric refrigeration prevails.* Miss I., aged 23—skin very cold to the touch in the last stage of the black vomit. Died at 10 A. M., Sept. At noon air about 80°; two hours after death—axilla 5 m. 102°; vagina 5 m. 104°—5 m. 105° —10 m. 105°; axilla 5 m. nearly 104°—10 m. the same. Three hours after death, vagina 5 m. 104°; epigast. 7 m. 105°—3 m. falling—8 m. 104°; vagina 5 m. 104°, falling; rectum 5 m. 104°. Four hours after death, epigast. 5 m. 104°—3 m. 104°; vagina 5 m. 104° nearly. Six hours after death, axilla and thigh each 100°; epigast. 103°.

*The thigh the hottest, but cools pari passu with the centre, contrary to the law of refrigeration in dead matter.* F. L., born in, and last from France, aged 58; resident two months; sick eleven days; died Sept. 13th, at 5 P. M.; room 86°—10 m. after death, axilla 102°; knees by contact, without incision, 4 m. 102°; rectum 5 m. 104°; axilla 5 m. 104°; thigh 3 m. 104°—6 m. 106° and over; epigast. 4 m. 106° and over; thigh 3 m. nearly 108°—3 m. 108° fully—5 m. fell to 104°; left lung 5 m. 104°—right, in 5 m. over 103°; the other thigh 3 m. 104—2 m. 104°—3 m. 104°; base of the right lung 103°; thigh (old incision) 103°; epigast. 103°. Nineteen hours after death; room 86°; epigast. 89°; thigh 89°; left chest 88°—right 89°; middle of the arm 89°; calf of the leg 86°, exactly agreeing with the air. This subject, before rigidity set in, performed supination, pronation and flexion most beautifully. When the arm was extended at a right angle with the trunk, a blow over the biceps, not sufficient to injure the living, caused the fore-arm to bend, carrying the hand to the chest repeatedly.

*Calorific focus in the epigastrium; in two hours the thigh exceeds the heart in temperature more than* 4°. F., born in Cincinnati; steam boatman; aged 24; resident eleven days; treated with foot-bath, cups, enemata, sponging with hot brandy, carb. ammon., sinap., camphor, phos. calcis, cold to the head, arteriotomy. Aug. 29, 11 A. M., air 83°; sick eight days; hand 15 m. 104°; axilla 10 m. 105°. Sept. 1st, 1 P. M., room 84°. Dead half an hour; axilla 5 m. 101°—2 m. 104°—2 m. 105°—2 m. over 106°—2 m. same; thigh 5 m. over 106° —2 m. 107°—2 m. 108°—2 m. 107°; axilla 5 m. 107°, left epigast. 3 m. 109¼—2 m. 109°; right epigast. 5 m. 109°—at sundry places by perforating the linea alba, 106°; thigh 5 m. over 106°-5 m. same; base of the lungs and heart each 102°—an hour after removing the abdominal *viscera* the thigh gave 33° of Reaumur, or more than 106° of Fah. [A blow given with the flat side of the hatchet, over the biceps, caused the hand to be placed against the ear; a second blow carried it to the nipple; a third brought it to the perpendicular; a fourth gave but a feeble motion to the hand, without elevating the fore-arm. The blows had been purposely severe; the contractility was killed; the muscle became inelastic or doughy, receiving the impression of the instrument from the last stroke. An hour after the other arm was very contractile.]

NOTE.—*Post-mortem* muscular contractility, and its very curious laws, I accidentally noticed several years ago; its production, duration and decline were all new to me. I have sought in vain for any satisfactory record of these phenomena, except in connection with galvanism. Had I time, I could turn to my MS. volumes containing some cases wherein this property continued six to seven hours after death, and hours after amputating the shoulder, with all its muscles—the fore-arm flexing so violently as to turn the shoulder over, causing the deltoid to rest on the table, instead of being uppermost, as it was before the muscular action. It is my intention to give an outline of this subject, as soon as convenient. I certainly do not wish to indulge any unwarrantable pretensions to discovery. Perhaps every body knows the facts to which I allude. But it is strange that our teachers do not show these experiments to students; they are infinitely preferable to the mummery of galvanism. A blow with the professor's hand will explain more of muscular contraction, than a thousand words. The muscles may all be even severed except the flexors of the fore-arm—the latter being dissected bare—the whole process and duration of contraction pass before the eyes, even after the amputation of the shoulder, proving that the spinal marrow is not necessary "to the contractile function of the limbs," as said by Dr. Hall.*—Generally, muscular rigidity (*rigor mortis*) first begins, and first ends, in the neck; *post-mortem* refrigeration takes place first in the head—these, with many other phenomena, incline me to think that soon or late it will be admitted that death, usually, if not always, begins in the brain, and that the body dies downward along the centre, and then outwardly, notwithstanding all that the illustrious Bichat has said of death beginning in the heart and lungs. This biceps muscle outlives any of the organs called vital.—*Bost. Med. & Surg. Jour.*

* Vide the able work of Dr. M. Hall, New Syst., p. 42.

